# Toward Machine-Learning-Based Decision Support in Diabetes Care: A Risk Stratification Study on Diabetic Foot Ulcer and Amputation

**DOI:** 10.3389/fmed.2020.601602

**Published:** 2021-02-18

**Authors:** Zeinab Schäfer, Andreas Mathisen, Katrine Svendsen, Susanne Engberg, Trine Rolighed Thomsen, Klaus Kirketerp-Møller

**Affiliations:** ^1^Department of Computer Science, Aarhus University, Aarhus, Denmark; ^2^Research Unit for Mental Public Health, Department of Public Health, Aarhus University, Aarhus, Denmark; ^3^Steno Diabetes Center, Copenhagen, Denmark; ^4^Department of Chemistry and Biosciences, Center for Microbial Communities, Aalborg University, Aalborg, Denmark; ^5^Life Science, Danish Technological Institute, Taastrup, Denmark; ^6^Department of Dermatology, Venereology and Wounds, Copenhagen Wound Healing Center, Bispebjerg Hospital, Copenhagen, Denmark

**Keywords:** risk assessment, amputation, prediction models, cohort analyses, diabetic foot ulcer

## Abstract

Diabetes mellitus is associated with serious complications, with foot ulcers and amputation of limbs among the most debilitating consequences of late diagnosis and treatment of foot ulcers. Thus, prediction and on-time treatment of diabetic foot ulcers (DFU) are of great importance for improving and maintaining patients' quality of life and avoiding the consequent socio-economical burden of amputation. In this study, we use Danish national registry data to understand the risk factors of developing diabetic foot ulcers and amputation among patients with diabetes. We analyze the data of 246,705 patients with diabetes to assess some of the main risk factors for developing DFU/amputation. We study the socioeconomic information and past medical history of the patients. Factors, such as low family disposable income, cardiovascular disorders, peripheral artery, neuropathy, and chronic renal complications are among the important risk factors. Mental disorders and depression, albeit not as pronounced, still pose higher risks in comparison to the group of people without these complications. We further use machine learning techniques to assess the practical usefulness of such risk factors for predicting foot ulcers and amputation. Finally, we outline the limitations of working with registry data sources and explain potentials for combining additional public and private data sources in future applications of artificial intelligence (AI) to improve the prediction of diabetic foot ulcers and amputation.

## 1. Introduction

Diabetes mellitus, a worldwide pandemic that is expected to rise to 700 million cases by 2045, is a serious challenge for both patients and healthcare professionals [1]. The alarming numbers in the prevalence of diabetes and the high social and financial costs associated with the disease indicate a pressing need for further improving effective control and prevention strategies [6]. Among the most disabling, complex, and costly complications of diabetes are diabetic foot ulceration (DFU) and amputation [2–5]. Various studies have investigated risk factors for developing DFU [7, 8]. Such studies have traditionally been conducted with controlled cohorts that allow for adjustment of the most likely confounding variables but often with study scale as the main trade-off. However, the abundance of digital medical data available today presents new opportunities to drastically increase the scale of future risk stratification studies and explore different risk factors. Moreover, through the combination of different data sources and incorporating risk analysis studies, it becomes more tractable to develop decision support based on machine learning, e.g., to support early detection or monitoring of certain conditions.

In order to model a decision system that is able to assess the risk of developing DFU/ amputation, it is required to understand the best set of underlying features and how their combination can improve the prediction. In numerous studies, the impact of different socio-economic status of patients in relation to diabetes has been studied [9]. As an example, it has been shown that the income level is inversely correlated with diabetes (type 2) [10, 11]. In Cosgrove [9] and Pouwer et al. [12], the effect of stress, stressful life events, such as changes in family status, salary income, and working condition in relation to diabetes have been assessed.

In a study conducted by Hangaard et al. [8], a cohort analysis of 5,588 patients with type 1 diabetes and 7,113 patients with type 2 diabetes has been applied where risk factors for first-time foot ulcers have been shown using general clinical information that is already obtained during routine follow-ups. Among the risk factors were long diabetes duration, history of cardiovascular disease, decreased visual acuity, diabetic retinopathy, and self-reported neuropathic symptoms. Engberg et al. [7] also show being male, having type 2 diabetes, and smoking increase the risk of getting a recurrent foot ulcer in their cohort study with 780 people. In addition, they present that patients with neuro-ischaemic or critically ischaemic ulcers have a higher risk of developing recurrent ulcers compared to patients with neuropathic ulcers.

Sen et al. [13] used a meta-analysis approach, coalescing findings from multiple risk stratification studies specifically about amputation for patients with a diabetic foot infection. Based on a set of inclusion criteria, they included 25 articles in their study totaling 6,132 patients. Among the predictors for amputation in patients with diabetic foot infection were a previous history of amputation, peripheral arterial disease, retinopathy, osteomyelitis, and gangrene/necrosis. While most risk stratification studies largely confirm and report on similar factors, we are still to see larger studies on nationwide diabetes populations, which also include more factors about general socio-economic well-being.

We therefore assess the risk level of developing DFU/amputation and the mortality using the available socio-economic registry data and available medical registrations of patients. The purpose of this study is 2-fold. The first aim is to investigate risk factors and features based on a large readily available medical dataset, which can be used for early detection of patients who are at high risk of developing DFU or amputation. Among the significant risk factors are the traditional major complications of diabetes, disposable income, mental disorders, such as depression and dementia. While there are limitations with the precision of the available data, we believe that the volume of our study strengthens the findings that both confirm existing risk factors and indicate new features promising to be scrutinized further in the field. Accordingly, this part also serves to assess how existing large data sources can be used to assess known risk factors, explore new risk factors, and to understand the weaknesses of such data analysis. The second aim is to utilize machine learning (ML) approaches to predict the occurrence of DFU and amputation in order to assess the practical usefulness of risk factors based on general socio-economic information of patients and their medical history. Thus, the long-term goal of our work is to unfold the potential of using different sporadic health-care data sources which may be combined for better machine-learning-based decision support in the future.

## 2. Materials and Methods

### 2.1. Data Sources

**Figure 1:**
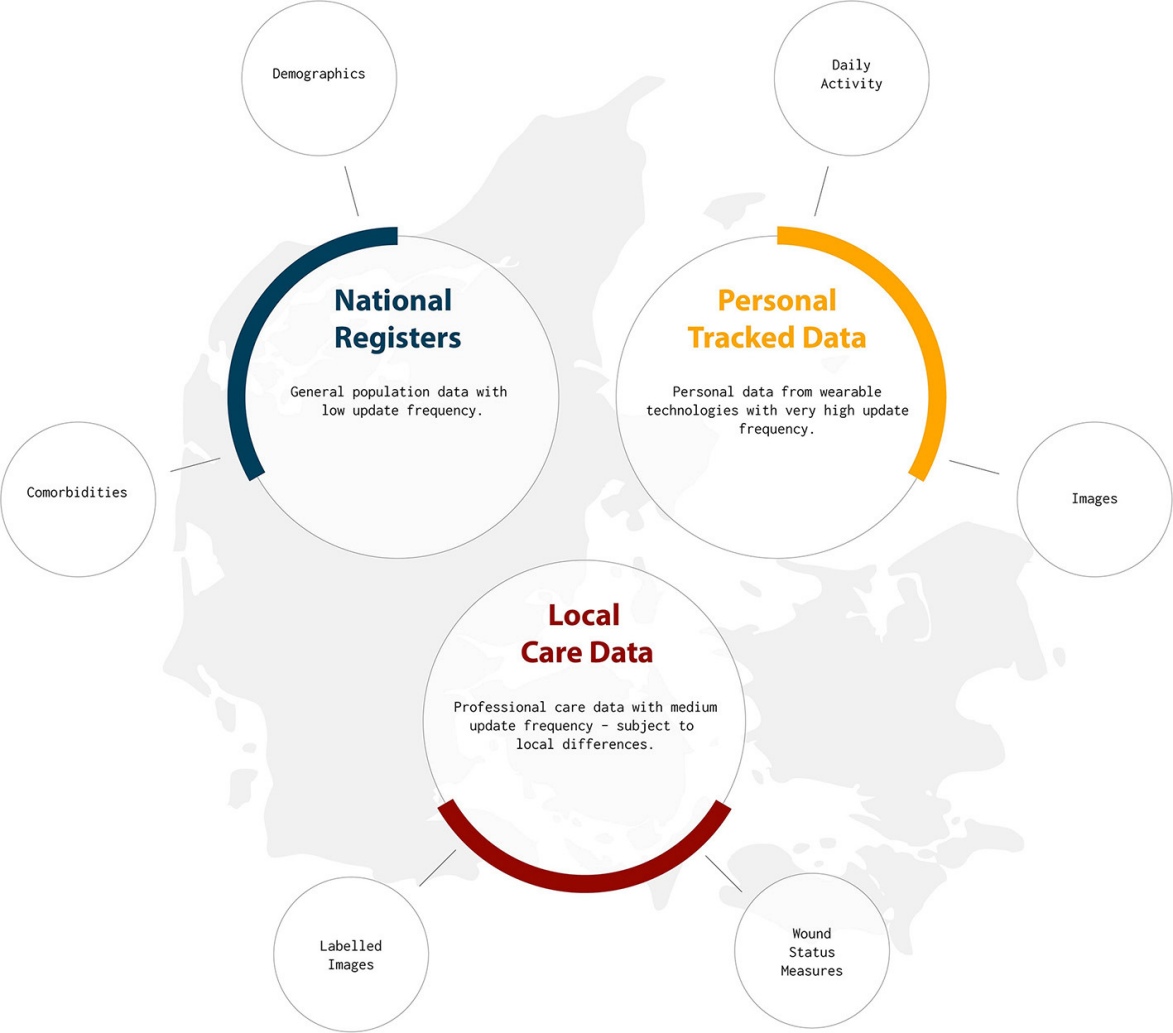
A general overview of existing diabetes related data sources in Denmark. The current study is based on data from national registry. Potential future data sources include the local care and personal tracked data.

Healthcare data is usually scattered throughout multiple systems maintained by different entities and each with a different level of detail and granularity. In Denmark, the data sources can generally be divided into three categories; the national registers, local care data, and personalized tracked data. This study relies on the national registers^1^, which are population-wide databases that historically have been established for administrative purposes, but they also serve as sources to compute general statistics about the entire population, e.g., to support policymaking. All data in the national registers are connected through a personal identification number (CPR) from the Danish Civil Registration System [14], which allows unambiguous linkage of the registers. The Danish Civil Registration System contains information about all residents in Denmark, including citizenship, birth, and family status. Among the more prominent medical register, are the Danish National Patient Register (LPR) [15] and the Danish National Prescription Registry [16]. Using all these national registry systems, we have access to diagnoses, treatments, procedures, and prescribed medications, as well as general socioeconomic information, such as salary, public welfare benefits, employment status, and addresses. Other different data sources, such as personal tracked data and local care data will be used for our future studies (see Figure 1). For more details see section 4.1.

### 2.2. Population Data Statistics

We conduct a population cohort study on Danish national registers with citizens born between 1900 and 1968, which includes the data from 3,500,877 citizens. We only have the available medical and socio-economic data from the years 2000 to 2018. The focus of our study is on the time period since the first onset of diabetes from 2000 to DFU and amputation. In cases where none of these events occur, we consider the death year or the end of the study being 2018. As we do not have access to the entire life history of the patients (only the period between 2000 and 2018), it is challenging to obtain the exact year of a diabetes diagnosis. Therefore, we assume the earliest recorded year of diabetes diagnosis in the period of 2000 to 2018 to be the onset of diabetes for the patients.

Among the full population, there are 247,208 citizens that have been diagnosed with any type of diabetes at some point. We exclude entries with incomplete data, as well as entries where general foot-ulcer, amputation, or death were registered before the diabetes diagnosis. Thus, in total, we consider a population of 246,705 patients with diabetes (among them, 531 patients are younger than 31 years old at the time of diabetes diagnosis, and we kept them in the dataset). Among the population of patients with diabetes, there are 13,695 registered cases of DFU and 7,540 cases of amputation. The median observation period for the patients with a registered DFU or amputation diagnosis (time period since diabetes year until DFU or amputation) based on our data is 8 years (mean of 7.74±5.8) and 6 years (mean of 6.8±5.29), respectively. The median follow-up duration for patients without DFU or amputation complication is 9 years (mean of 9.4±5.8 years). It is important to state that because of a lack of access to the entire patient history described earlier, the reported duration is not accurate and subject to bias.

All codes for diagnosis of diseases are based on the *international classification of diseases ICD-10* [17]. Codes corresponding to different medications are based on the ATC/DDD index [18]. For example, to identify patients with diabetes, we use all diagnosis codes from the diabetes diagnoses tree as well as codes for relevant antidiabetic medicine. In Denmark, we further use a coding scheme named SKS, which incorporates ICD-10 but extends certain parts of the tree. SKS is governed by The Danish Health Data Authority and is used by all public healthcare institutions. This is in particular useful for identifying patients diagnosed with DFU. A recent paper has described how ICD-10 codes provide poor positive predictive values for DFU [19]. In their study, they use the diabetes codes E10.5–14.5, which cover both diabetic gangrene, peripheral angiopathy, and ulcer for different diabetes types—i.e., these codes do not only cover DFU and therefore they are subject to false positives. The SKS coding scheme further subdivides E10.5–14.5 into A-D, which means that the codes E10.5B–E14.5B that have been used for this study are specifically for DFU. All codes used in this study have been included in the section 1.1 in Supplementary Material. We discuss some of the limitations with regard to the coding system and the fixed time period in section 4.1.

Using the available population data, we extract the following features and information: the presence of any of the following medical conditions before the event of interest (i.e., foot ulcer or amputation); and cardiovascular-related disorders, neuropathy disorders, peripheral artery diseases, hypertension, hyperlipidemia, chronic renal disorder, urinary tract infection, retinopathy, mental disorder, depression, dementia with Alzheimer, and nervous system disorder (see Appendix in Supplementary Material for the corresponding ICD-10 codes). The socio-economical features used are as follows:

- The past medical history of the patients
- The average number of Statins and diabetic-related medication prescribed annually
- The number of changes in the home address registry
- Household disposable income
- Age, gender, and Ethnic background (Danish and immigrant citizen)
- The number of changes in the family status
- Effect of different medical conditions on the risk of DFU/amputation.

We use the Cox proportional-hazard model [20] and Aalen Johansen model to analyze the risk effect of the listed covariates in the study. The event of interest considered is the occurrence of amputation and foot ulcers. It is important to state that in the available database, 30% of amputation registries have been recorded in the same year as the foot ulcer (42% of people who got amputations had no foot ulcer registration code). From 2,402 patients that have foot ulcers and amputation registered in the same year, in 75% of the cases, the events are registered with a difference of 2 months or less apart. We believe that this could be due to inaccurate recording of the diagnoses or significant delays in diagnosing foot ulcers. Despite an overlap in the coding registration, we analyze the two events of foot ulcer and amputation separately, as amputation occurs as a consequence of a delayed treated foot ulcer.

### 2.3. Statistical Models

The survival function *S*(*t*) is defined as the probability of a person not experiencing the event of interest until a specified time *t*. It is denoted as *S*(*t*) = *Pr*{*T* ≤ *t*} = 1−*F*(*t*) where *T* is a continuous random variable for a person's survival time, *t* is a specific value of interest for *T* and *F*(*t*) = *Pr*{*t* < *T*} is the cumulative distribution function of the random variable *T* (see chapter 1 of [20]).

Given that an individual has survived up to time *t*, the hazard function (also known as the condition failure rate) *h*(*t*) is the instantaneous potential that the event of interest occurs at time *t*. Mathematically the hazard function is defined as

(1) $$\begin{array}{l} h\left(t\right)\;:=\;\underset{\Delta t\to 0}{\lim}\frac{P\left(T\leq t+\Delta t|T\geq t\right)}{\Delta t}, \end{array}$$

This definition implies that *h*(*t*) ≥ 0. Note that *h*(*t*) is not a probability and in particular has no upper bound.

Using the same terminology as above, the Cox PH model is essentially a linear regression to the (log) hazard function. The Cox PH model parameterizes the hazard function as follows:

(2) $$\begin{array}{l} h\left(t,X\right)=h_{0}\left(t\right)\exp\left(\underset{i}{\sum }\beta_{i}X_{i}\right) \end{array}$$

where *h*_0_(*t*) is the *baseline hazard function* and the β_*i*_ correspond to the coefficients in a linear combination of explanatory variables *X*_*i*_.

The Cox model expresses the hazard at time *t* for a person with given specific explanatory variables (covariates). In this model, the baseline hazard function depends on the time *t*; however, the explanatory variables are not time-dependent. The coefficients β_*i*_ are computed through maximum likelihood estimation. For more details regarding the model and estimation techniques, see chapter 3 of [20].

The *hazard ratio* is defined as the hazard rate of a person with a set of specific predictor variables divided by the hazard for a different individual. That this ratio is a constant is the basic assumption of the Cox model. The expression exp(β_*i*_) hence corresponds to the hazard ratio of a patient with feature *X*_*i*_ = 1 vs. a patient with *X*_*i*_ = 0 and with exactly the same features apart from that.

#### 2.3.1. Extended Time-Varying Cox (TVC) Model

Unlike the basic Cox PH model, the TVC model assumes that the indicator variables *X*_*i*_(*t*) depend on the time *t*. This leads to the following expression for the hazard function.

(3) $$\begin{array}{l} h\left(t,X\right)=h_{0}\left(t\right)\exp\left(\underset{i}{\sum }\beta_{i}X_{i}\left(t\right)\right) \end{array}$$

The regression coefficients β_*i*_ are computed in a similar way as for the basic Cox model using maximum likelihood estimation. For a complete description of the methodology, see chapter 6 of [20]. We used the lifelines package, an open-source python library to conduct our survival analysis studies.

In our application, the variables *X*_*i*_(*t*) track the onset time of a particular disorder. Before the onset of a disease we set *X*_*i*_(*t*) = 0 and afterwards, until the end of the study we have *X*_*i*_(*t*) = 1. We also stratify the population-based on sex and age where it is assumed that the baseline hazards are distinct (chapter 6, [20]). In addition, we apply the Mann-Whitney *U*-test to gauge which features are significant contributors to the risk of developing complications, such as foot ulcers or amputation.

#### 2.3.2. The Aalen Johansen Model

The Cox PH model estimates the conditional probability of developing DFU or amputation, i.e., the probability assuming zero mortality. This model is mainly useful for a qualitative assessment of the increase in risk due to other complications. On the other hand, the model slightly overestimates the true probability of developing DFU or amputation. We, therefore, used the Aalen Johansen Model to estimate the probability of developing DFU or amputation, while accounting for mortality, that is to take into account *competing events* that may preclude the event of interest from occurring.

The Aalen Johansen model is a non-parametric approach for computing the cumulative hazard rate and is expressed as *F*(*t, j*) = *P*(*T* ≤ *t, J* = *j*) where *T* is the time since origin to the event of interest or competing for an event and *J* denotes the type of event (see [21] for more details). Due to the high average age of our population and high mortality during the study period, we compute the cumulative density for the following two cases:

1. The risk of getting DFU/amputation
2. The mortality

We apply a separate model for female and male patients at different age groups. For comparison, we also produce a baseline model that considers the total population with any type of medical condition for the specific age group.

### 2.4. Applying Machine Learning Methods

**Figure 2:**
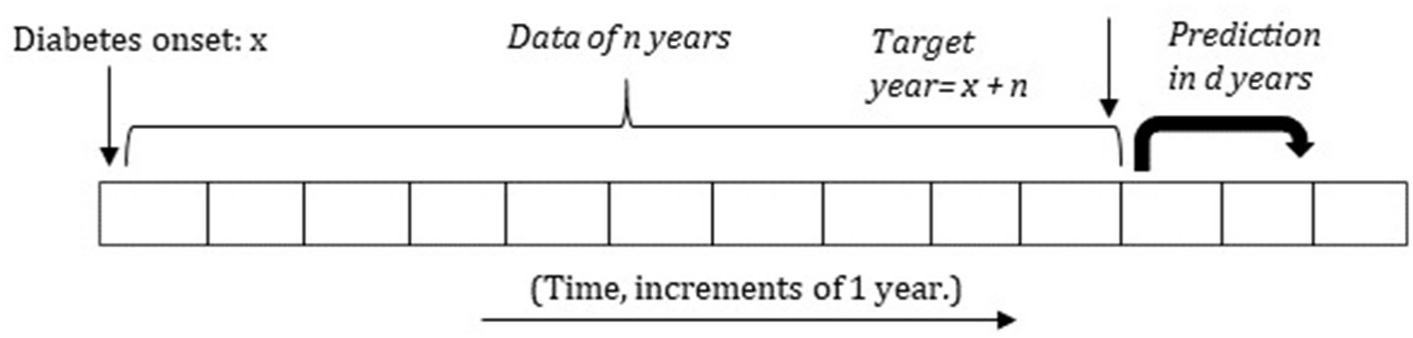
Structure of using the past data for predicting the future occurrence of amputation or foot ulcer in the next *d* years (setup 2). The variable n is set between 3 and 11 years.

After assessing the different risk factors, we apply machine learning to predict the occurrence of DFU/amputation at different time-intervals. As duration since diabetes to DFU/amputation is a key element in our study, we divide the data as follows: For a fixed number of years *n*, ranging from *n* = 3 to *n* = 11, we first get the data from diabetes onset until *n* years after. This is referred to as the target year. We remove the patients that die within this period (where they have less than *n* years of data available) unless they developed DFU/amputation within this time period. Figure 2 presents a small illustration of the timeline and its structure.

We use two model setups. In setup 1, we divide the remaining patients into two classes. The first class consists of all patients who developed DFU/amputation within the target year. The second class consists of all patients who did not develop any DFU/amputation for at least the first *n* years after the onset of diabetes. The goal is to classify between the two classes, which serves to demonstrate that the two classes can be distinguished at all based on the provided features. For each class, features are based on data from diabetes onset until either the occurrence of DFU/amputation or until the target year. Thus, this setup models whether patients get DFU/amputation at all given their medical history up until a maximum of *n* years.

For setup 2, we take the information of patients for the first *n* years since diabetes onset and predict whether they will develop DFU/amputation within the next *d* years. Thus, in this setup patients with a similar history of no DFU/amputation in the first *n* years of their diabetes history are compared and it is specified for which period prediction is done.

Setup 1 has less practical value compared to setup 2, as patients are compared based on ideal points in time of their diabetes lifespan, i.e., using all information right up until the occurrence of DFU/amputation or until the end of the fixed study period. Setup 2 more directly models predictive potential in real-life settings, but it is also much more restrictive in that it only compares patients with similar time periods of no DFU/amputation. Thus, in reality, the true predictive potential of the data in this study may lie somewhere in between the two model setups.

In both cases, for scalar features, such as the number of address changes, average family income, and medication intake, the yearly average is used. In order to make the intake of medications comparable between different patients, the data is normalized based on the duration time since diabetes onset to the occurrence of DFU/amputation or end of the study for the patient (which is either death or end of the study in case of no event observation). Boolean features, such as the occurrence of any medical condition are only taken into account if they occur before and including the target year and before the development of DFU/amputation. In both setups, logistic regression (LR) and random forest (RF) classifiers are used and compared against the results of a baseline classifier.

## 3. Results

In Table 1, we present an overview of data along with the statistics of different medical conditions. Results corresponding to some of the entries in the table are based on the quality of data which we discuss in the limitation section. In the following, we present the risk analysis of the medical conditions outlined previously and the socio-economic factors.

**Table 1:** Data characteristics of the patients included in the study.

	**Patients with diabetes (*****N*** **=** **246,705)**		
	**No complication**	**Foot ulcer**	**Amputation**
	**(*n* = 229,681)**	**(*n* = 13,695)**	**(*n* = 7,540)**
	***n* (%)**	***n* (%)**	***n* (%)**
**Gender**			
Male	131,852 (57.4)	9,520 (69.5)	5,439 (72.1)
Female	97,829 (42.6)	4,175 (30.5)	2,101 (27.9)
**Age**			
<31	491 (0.2)	37 (0.3)	14 (0.2)
31–54	62,728 (27.3)	4,382 (32.0)	1,979 (26.2)
55–63	60,000 (26.1)	3,682 (26.9)	1,952 (25.9)
64–71	52,007 (22.6)	2,888 (21.1)	1,753 (23.2)
72–98	54,453 (23.7)	2,706 (19.8)	1,842 (24.4)
**Ethnic background**			
Danish	204,765 (89.2)	12,843 (93.8)	7,185 (95.3)
Immigrant	24,560 (10.7)	829 (6.1)	347 (4.6)
Descendant	356 (0.2)	23 (0.2)	8 (0.1)
**Family disposable income**			
DKK <179,001	74,434 (32.4)	5,343 (39.0)	3,634 (48.2)
DKK 179,001–292,000	76,713 (33.4)	4,639 (33.9)	2,446 (32.4)
DKK >292,000	78,277 (34.1)	3,691 (27.0)	1,448 (19.2)
Bone fracture	55,904 (24.3)	3,565 (26.0)	1,628 (21.6)
Cardiovascular disease	53,410 (23.3)	11,046 (80.7)	5,502 (73.0)
Chronic kidney complication	18,483 (8.0)	1,894 (13.8)	1,087 (14.4)
Dementia	1,606 (0.7)	40 (0.3)	22 (0.3)
Depression	606 (0.3)	25 (0.2)	7 (0.1)
Neuropathy	11,119 (4.8)	2,042 (14.9)	1,093 (14.5)
Retinoathy	9,707 (4.2)	961 (7.0)	467 (6.2)
Hyperlipidemia	4,320 (1.9)	207 (1.5)	76 (1.0)
Hypertension	27,465 (12.0)	1,680 (12.3)	756 (10.0)
Mental disorders	17,364 (7.6)	1,015 (7.4)	432 (5.7)
Nervous system disorder	57,805 (25.2)	4,420 (32.3)	1,984 (26.3)
Periphery artery disorder	10,488 (4.6)	2,023 (14.8)	1,695 (22.5)
Urinary tract infection	21,208 (9.2)	1,302 (9.5)	620 (8.2)
Multiple unspecified complications	14,681 (6.4)	2,967 (21.7)	1,933 (25.6)

### 3.1. Risk Factors for Diabetic Foot Ulceration and Amputation

**Figure 3:**
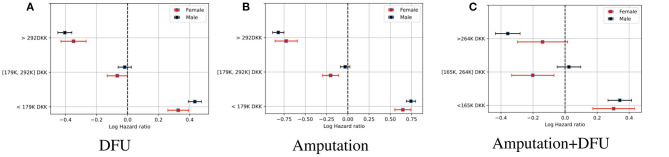
The log hazard ratio for household disposable income for patients with diabetes and foot ulcer, amputation, and those who have foot ulcer and amputation. The risk of DFU/amputation event is higher for the 33rd percentile. **(A)** DFU. **(B)** Amputation. **(C)** Amputation + DFU.

Figure 3 shows the log hazard ratio for average household disposable income for patients with foot ulcers, amputation, and those with amputations who also had foot ulcers. We group the data into three groups, which are split at the 33rd and 66th percentiles, respectively. A Cox PH model is applied for every percentile if the subject falls within that group.

**Figure 4:**
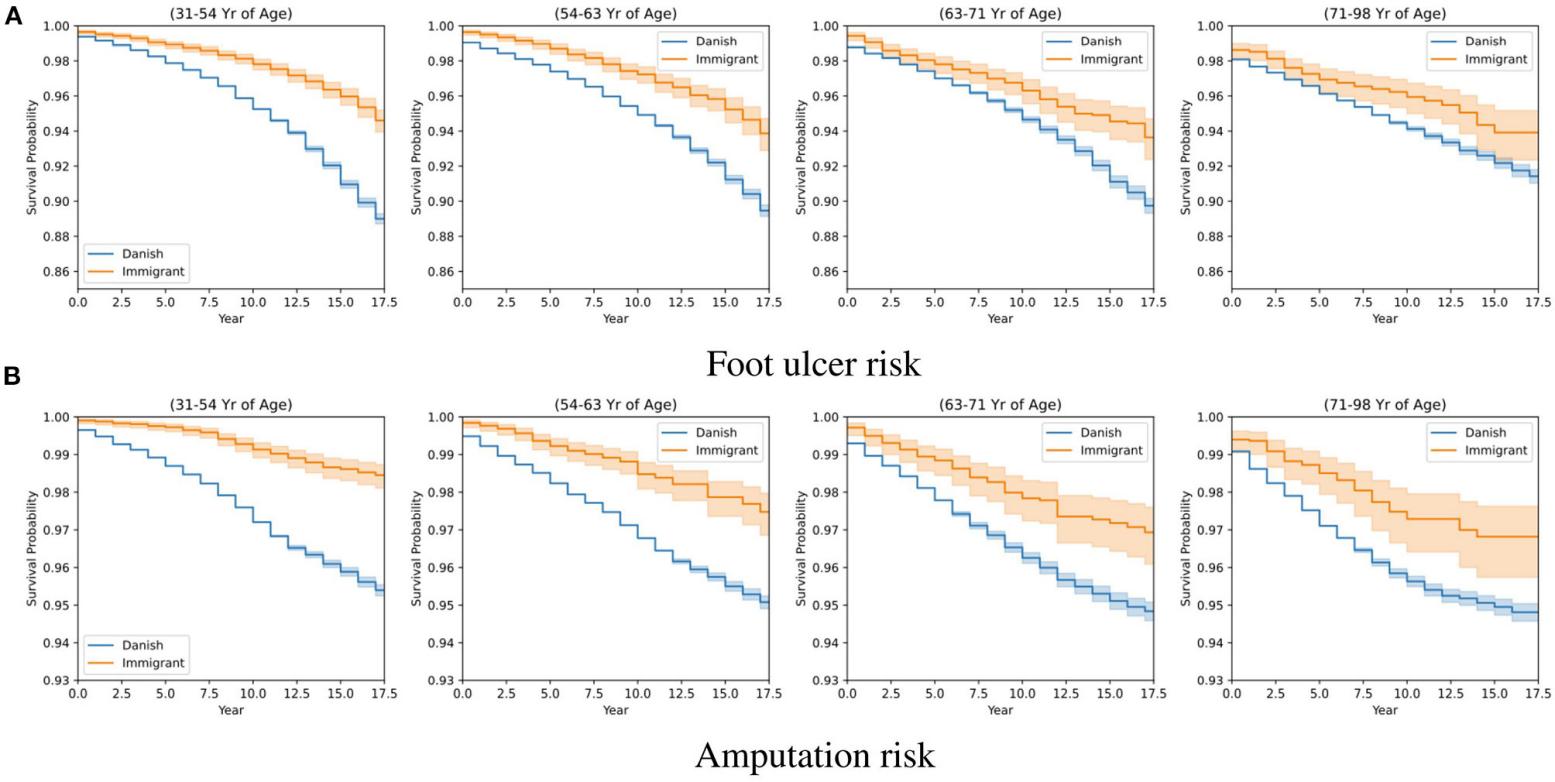
Kaplan-Meier estimates for different age groups and backgrounds and the corresponding risk levels for foot ulcer and amputation. **(A)** Foot ulcer risk. **(B)** Amputation risk.

The survival probability of DFU/amputation for a population of Danes and immigrants is included in Figure 4 where we apply a Kaplan Meier estimator at four distinct age groups for the Danish (blue line) and immigrant population (yellow line). The x-axis presents the number of years since diabetes onset, and the y-axis is the corresponding probability of surviving DFU (plot a) and amputation (plot b). The survival probability for the immigrants is slightly higher than the Danish population of patients. As we explain in the discussion, this can be related to a generally younger population of immigrants as well as a smaller sample size.

**Figure 5:**
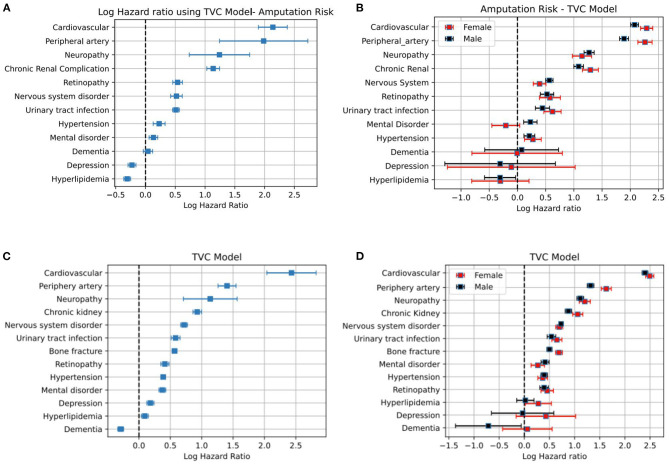
Plots **(A–D)** correspond to the log-hazard ratio of patients who get amputation and foot ulcer, respectively.

Figure 5 shows the hazard ratio for different medical conditions. The predictors for every medical condition are age, gender and the event of interest is the observation of DFU/amputation. The duration from diabetes until the occurrence of DFU/amputation has been corrected for individuals deceased during the study period. The study period for these individuals has been set to the date of death. In other cases, the study period is the end of the study in 2018 or the year in which the event has occurred. The models are stratified based on age and gender in plots (a,c) and applied separately for female and male populations as in (b,d). Results indicate the level of increase in the hazard rate for developing DFU/amputation for each medical category. The risk of amputation stratified based on major or minor cases and those who have foot ulcer have been included in Supplementary Figure 2 in Supplementary Material.

### 3.2. The Aalen Johansen Model on Medical Conditions and Mortality

**Figure 6:**
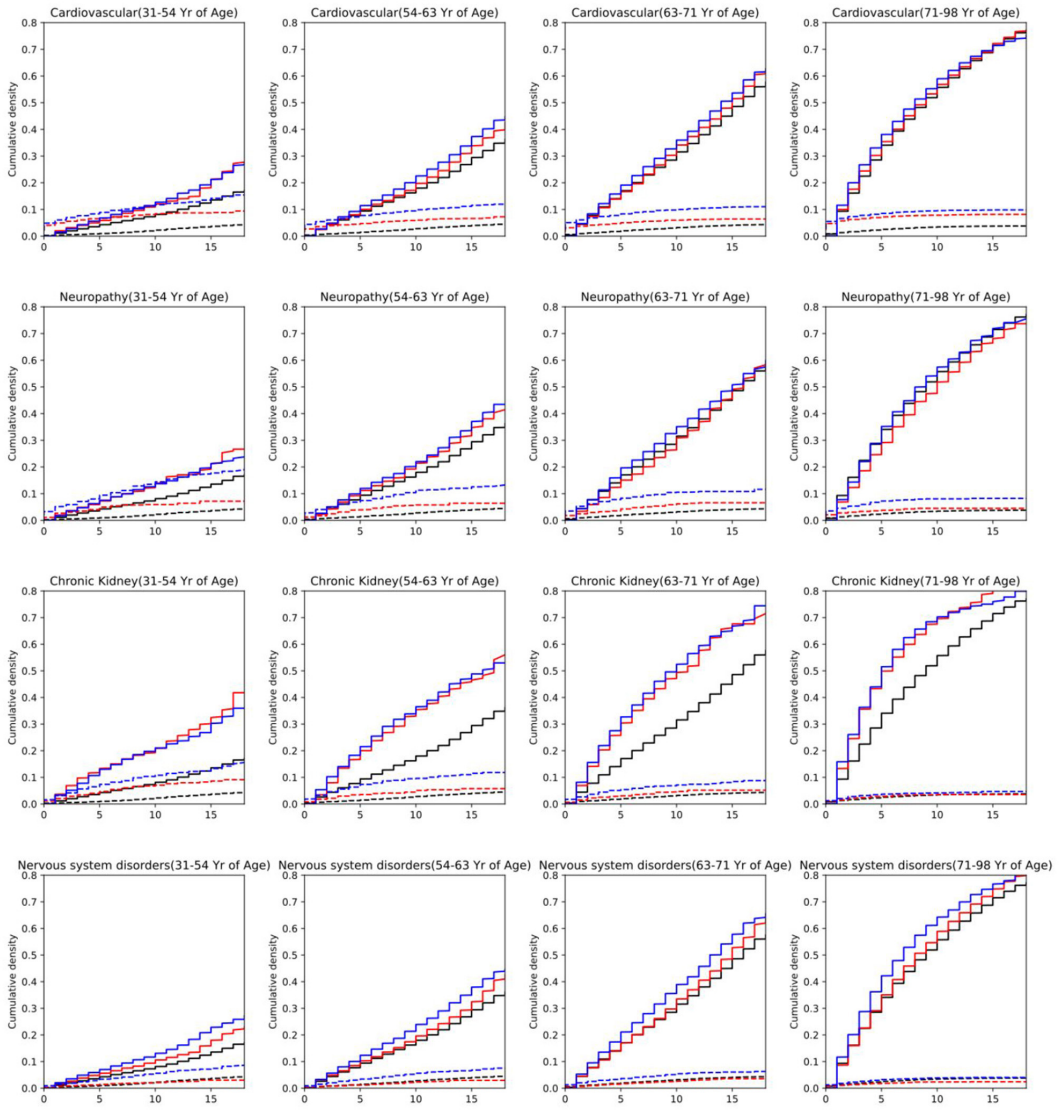
Risk of developing amputation or death for males and females at different age groups. Plots correspond to having diabetes and one of the medical conditions. Red (Female)/Blue (Male)/Black (Baseline) dashed lines: risk of developing amputation. Red/Blue/Black solid: (Female/Male/Baseline) risk of mortality. The x-axis denotes the number of years since diabetes onset. The baseline corresponds to the population of all people with diabetes.

In Figure 6 we compare mortality against the risk of DFU and amputation (see Supplementary Figures 3–5 in Supplementary Material for other medical conditions and the risk plots for DFU event). The risk of getting amputation and DFU for patients with cardiovascular complications is higher than the average risk of all patients with diabetes. A similar pattern is observed for neuropathy, chronic kidney, and nervous system disorder for both amputation and DFU events. In particular, the mortality risk for patients with diabetes and chronic kidney disorder is significantly above the average mortality risk of the population of all patients with diabetes. The risk values are observed to be slightly higher for men than female through all age groups.

### 3.3. Predictive Models

Based on the model setups described in section 2.4, we use the following features for LR and RF models:

1. Occurrence of any of the following medical conditions before and including the target year: neuropathy, retinopathy, hypertension, bone fracture, urinary tract infection, cardiovascular disease, chronic renal disease, hyperlipidemia, peripheral artery disorder, depression, dementia and Alzheimer, mental disorder, nervous system disorder, and DFU (for prediction of amputation)
2. Annual number of antidiabetic medicine, statin intake, and family disposable income up to and including the target year.
3. Annual average dose prescriptions of diabetes medication and statin up to and including the target year
4. Number of address changes up to and including the target year
5. Age (at the time of diagnosis of diabetes), Gender, family status, and background of patients.

**Figure 7:**
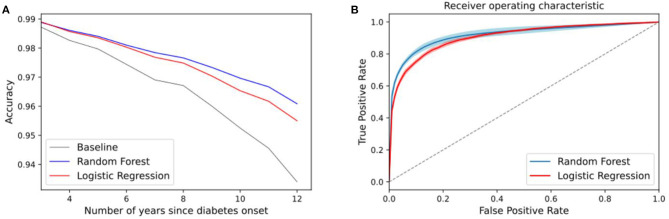
**(A)** Accuracy of the amputation classifier for different *n* years using a random forest and logistic regression. Baseline corresponds to a classifier that outputs no amputation. **(B)** Averaged ROC curves for *n* years. The diagonal line corresponds to a random classifier. Plots correspond to setup 1.

We randomly split the data into train and test sets and apply an RF model and an LR for prediction (25% of samples are used for the testing and 75% for training). The plot in Figure 7 shows the accuracy of the models vs. the baseline and the ROC curves. The baseline in (a) represents the accuracy of a baseline classifier, where the output is always no occurrence of amputation within *n* years since this is the largest class. The baseline in (b) corresponds to the diagonal line that has an area under the curve of 0.5 (a random classifier). A high value of true positive and zero false positive rates is the ideal situation of a perfect classifier.

**Figure 8:**
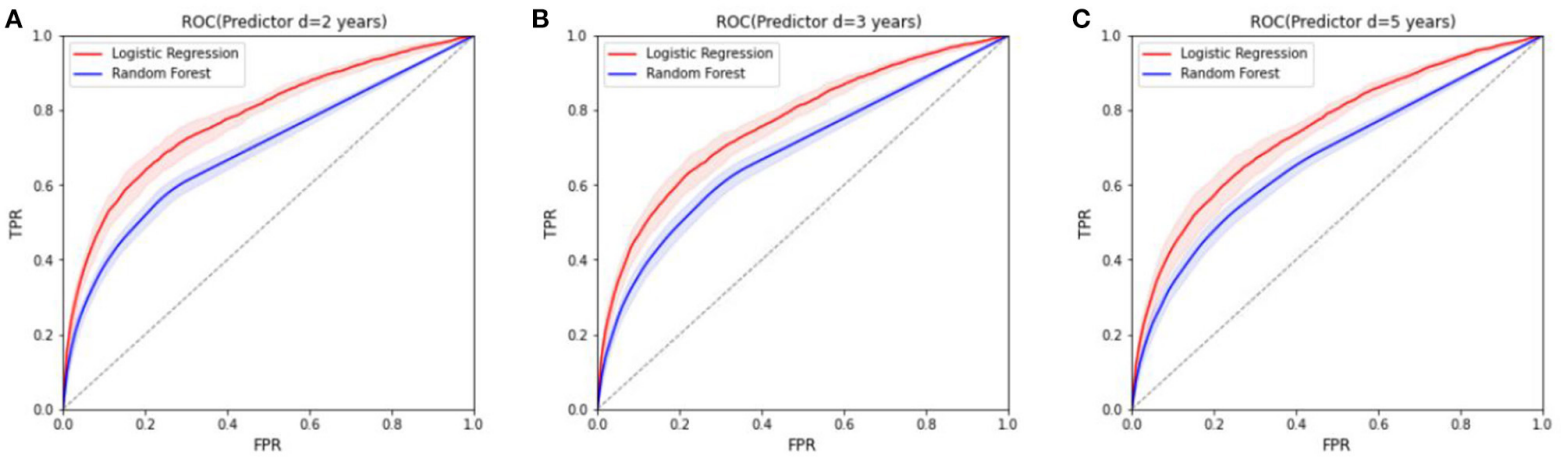
The ROCs for the prediction models of amputation in the next 2, 3, and 5 years, respectively (setup 2). The diagonal line is the baseline where predictions are random. **(A)** ROC (Predictor *d* = 2 years). **(B)** ROC (Predictor *d* = 3 years). **(C)** ROC (Predictor *d* = 5 years).

The plots in Figures 8A–C correspond to average ROC predictions of amputation occurrence at *d* = 2, 3, 5 years after the target year. We plot the mean and standard deviation of the ROC curves for different *n*. In addition the prediction results for DFU and amputation for those who have DFU have been included in Supplementary Figure 1 in Supplementary Material.

## 4. Discussion

Figure 3C clearly shows a higher hazard for patients with a lower household income. The finding have been confirmed and investigated before in several studies (see [22–25]). In this study, a similar outcome can be concluded with regard to the serious consequences of diabetes, namely DFU and amputation among lower-income families as well. The model is stratified on age for all three percentiles. By that, we mean that data is split into *m* smaller datasets based on age. Every dataset has a different baseline hazard rate; however, they all share the same regression parameters. The covariates that have been used for each dataset are the same, therefore the age is not shown separately in the results.

The plots in Figure 4 compare the survival probabilities between populations of Danes and immigrants over different years. Two factors that can explain the higher survival probability of the immigrant population over the Danish population can be explained in terms of the population size and the fact that the population of immigrants is younger than the Danish population in general (56.9±11.4 vs. 62.4±11.4 years old). As the population of immigrants is smaller as well, we split the data into four age groups and fit a separate model for each category. The number of Danes and immigrants for each age group is (56,277, 11,282), (57,447, 6,969), (51,463, 4,233), and (55,141, 2,986), respectively. We did not include the population of descendants in the study since the sample size is small and is subject to high variation. As the age increases, the standard deviation in the estimated survival probabilities increases, potentially narrowing the gap between the two different population backgrounds.

Beside the socioeconomic factors, the assessment of the risk of mortality and DFU/amputation for different medical conditions can be summarized as follows: diabetes patients with cardiovascular disorders, peripheral artery, neuropathy, and chronic renal complication are among populations with a high risk of developing DFU and amputation (see Figure 5). Similar results are obtained for major and minor amputations (see Supplementary Figure 2 in Supplementary Material). The high standard deviation for some cases in Supplementary Figure 2 in Supplementary Material is due to the relatively small population sizes because of stratification. This is more pronounced for depression, mental disorder, and dementia where it becomes difficult to report significant results. We used a separate Cox model for each medical condition to avoid the issue of confounding variables. This means that we cannot account for interactions between different risk factors. We leave such a study for potential future work.

As the age group increases in Figure 6 (Supplementary Figures 3–5 in Supplementary Material) and the duration since diabetes onset, the mortality risk increases significantly above the amputation risk for most medical conditions. However, in the lower age group, for cardiovascular and neuropathy, the risk of amputation is higher than the mortality risk at the beginning, and above the baseline for the general population of diabetes. This is highly expected as peripheral neuropathy is one of the contributors to DFU occurrence. This consequently increases the risk of amputation.

After assessing different risk factors, we use ML in two setups to evaluate the predictive value of the features with regard to DFU/amputation. The first model is set up to distinguish between two classes of people w/o amputation, we show the accuracy results for different target years in Figures 8A,B. The plots show the following: in (a) we exclude the first 2 years and last 6 years since the number of classes is significantly unbalanced. The number of people who get amputation only a year or two after diabetes onset is very low, therefore the baseline classifier has a high prediction accuracy (slightly above 97% accuracy). This number reduces as the number of years increases and the classes become more balanced. The lowest accuracy corresponds to within 10 years after diabetes onset using the logistic regression model which is at 95%, about 4% above the baseline predictions. Plot b corresponds to the ROC curves, which is independent of the class sample size.

The ROC curves of predictive models in Figure 8 for different *d* values (results have been averaged over *n*) illustrate the preliminary results of using the registry information. It is a more challenging task to predict the occurrence of DFU/amputation in the next coming years, based solely on the extracted socioeconomic features and past medical conditions. Therefore, we clearly observe a lower performance in the ROC curve in comparison to the classification task. Although the features that have been used in the study can be used for prediction models (based on the classifier results), they are not sufficient enough for accurate prediction of DFU/amputation. More detailed features and a higher time resolution are required for better predictive models.

### 4.1. Limitations and Strengths

Doing a study on existing medical data comes with added uncertainty as we have little control over the data collection process. In our study, we rely entirely on data from various national registers, thus the existing coding is used to identify all conditions. Although the coding is done by professionals, it may differ across regions, hospitals, and doctors subject to different coding preferences. While we do account for different ways of coding, e.g., diabetes and DFU, we may not have included all ways. This is also true for other diabetes-related comorbidities. As a result of the existing coding practice, certain complications have a low representation in the data used for the analysis. For example, one would expect that most patients suffering from DFU also suffer from neuropathy or some form of lowered nerve functioning in their feet. However, in the data of this study, only 14.9% have been diagnosed with neuropathy prior to DFU. This is in part a result of poor coding practice and the fact that data is not available for the entire lifespan of the participants. Some doctors may even use a single code named “diabetes with multiple complications” for patients suffering from more than one diabetes complication, which means it is impossible to distinguish exactly which complications are present after the fact. Another reason for the low prevalence of some conditions is the fact that only complications identified prior to DFU or amputation are included since the goal was to assess the practical usefulness of the available data for prediction. In several cases, complications are diagnoses at the same time as DFU, thus omitted in our analysis.

In addition to missing cases, we also expect some false positives in the data. Christensen et al. [19] recently described how using ICD-10 codes to identify DFU will give several false positives, as the current codes cover more diagnoses than just DFU. However, in our study, we use the Danish SKS coding scheme that, besides the ICD-10 codes, for example have specific codes for DFU (see section 2.2). Consequently, our results should contain fewer false positives compared to the results presented by Christensen et al. [19].

Another limiting factor in our study is the fact that we do not have medical and socio-economic data for the entire lifespan of the patients, since we only have data from 2000 until 2018. Thus, there are certain features that cannot be reliably estimated. For example, we are unable to compute whether a DFU diagnosis is a first-time incident or a recurrent one. Therefore, our study focuses on DFU occurrence in general, which means that we do not consider factors that are specific to first time or recurrent incidents. For instance, prior foot ulceration would likely be a strong predictor for recurrent ones. If we focused only on the recurrent DFU, we would limit our dataset significantly. In the same way, earlier diabetes diagnosis before 2000 cannot be ascertained, and this introduces a bias in the estimation of the target year.

The overlap between the foot ulcer registration year with the amputation registration year is also a disadvantage of the study, which further reveals the shortcomings with registry data in general. As mentioned previously in section 2.3, for patients with both amputation and foot ulcers, more than half of the cases occur in the same year. The separate analysis of the two events did not yield significantly different results due to the high overlap. This in particular signals a general delayed diagnosis of a foot ulcer or delayed registration of codes, which makes it challenging to design sensible predictive models for such conditions with register data alone. Despite the limiting factors of the data, the size of the dataset is a major strength. In fact, several of the factors that we show to significantly increase the risk of DFU/amputation replicate findings from other smaller studies, implying that the volume of the data mitigates some of the limitations. Assessing the usefulness of the available medical data for prediction purposes is therefore still very relevant in order to inform future studies and eventually practical applications.

#### 4.1.1. Future Work

**Figure 9:**
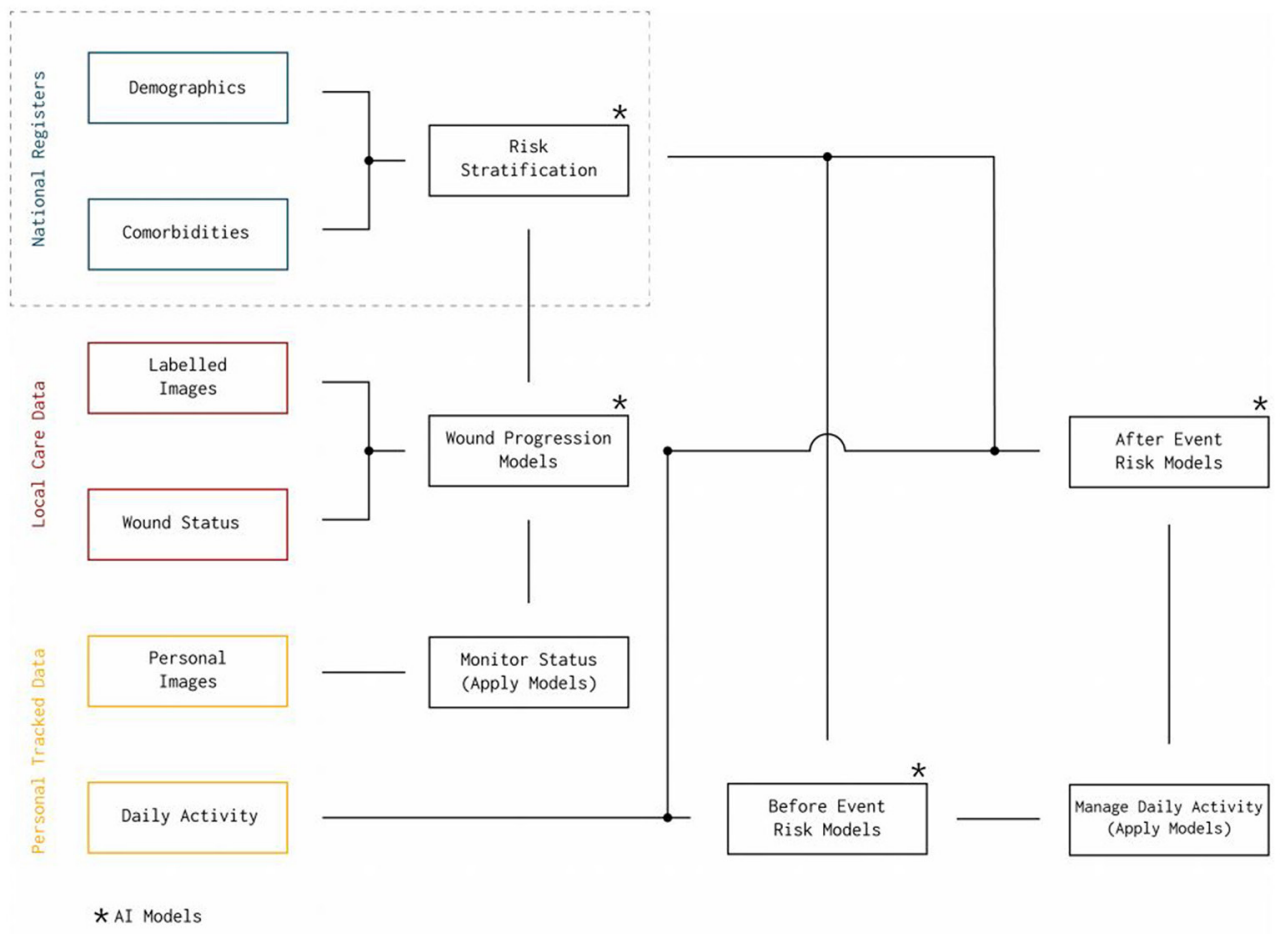
The flowchart-like graph depicts a potential future interplay between different data sources for application of AI in the domain of diabetic foot care.

Figure 9 depicts a potential future interplay between different data sources and different applications of AI that we aim to pursue in the upcoming studies. It highlights how the promised value of AI may be unlocked by combining existing data repositories with detailed quantified self data (personal tracking data), and how the result of one analysis can support the development of new and more advanced analysis models. The upper part of Figure 9 outlined by the dashed box represents the work on risk stratification presented in this paper. While risk stratification in itself is useful to learn about diabetes and its complications, the results from such an analysis can also directly be used in more precise and timely risk models that combine results from large cohort studies, with more detailed data about the everyday life of patients. This data could be provided by local care personnel and the patients themselves (see Figure 1).

One example is the opportunity to build machine learning models with existing wound image data and labels describing the current state of the wounds. Image recognition for diabetic feet is already an active field of research in which problems, such as wound segmentation [26, 27], ulcer detection [28], and recognition of ischemia and infection [26, 29] have been investigated. Thus, the next step may be to combine such methods with general risk factors to develop better models that are able to automatically label new images with information about the state of the wound progression. Such a wound progression model would be directly applicable in new tools both for patients and for doctors - patients would be able to self-monitor their wounds and doctors would be able to get detailed wound history that could support better medical advice.

Going even further, risk stratification and wound progression can serve as input to fine-grained risk models, both for predicting certain events and for predicting additional complications once an event has occurred. For instance, patients with slow healing progress may have a higher risk of getting an amputation, i.e., a wound progression model may be used to generate features for a model that tries to predict amputation. The presented flowchart is merely one suggestion of how we may combine detailed personal activity data with general risk factors and wound progression information to develop better prediction models in the area of diabetes. Still, there is much research needed to utilize the current data sources for accurate and reliable models that can be used for prediction of diabetes complications.

## 5. Conclusion

The risk assessment of different medical and socioeconomic features indicates that there is a high hazard of developing DFU or amputation for patients with diabetes and cardiovascular, peripheral artery disease, neuropathy, and chronic renal complications. It is also observed that mental disorders can slightly increase the risk; however, this is subject to more investigation as the category of mental disorders that have been used in this study covers a broad range of diagnoses. Family disposable income is inversely correlated with the risk of DFU and amputation. We did not find any significant correlation between DFU/amputation occurrence and the number of changes in the family-status or changes in the number of the home registry. However, the type of transitions in the family status, along with possible registered medical disorders are part of future investigation to single out different stressful events. Based on medical condition history and socioeconomic features, we were able to distinguish between patients with diabetes w/o DFU/amputation with an accuracy above the baseline over different years. This shows that in principle the two populations can be distinguished based on the available features. However, the results corresponding to the prediction of DFU/amputation in the next years require improvements. This has to be done by applying more feature engineering and obtaining more information on the medical and physiological history of the patients. Changing the timeline of the study is also one of the main factors for modeling future events. In addition, utilization of different data sources, such as images and user tracking data will allow for models that utilize data closer to the time of interest, thus allowing for more timely predictions that may better support current medical practices.

## Data Availability Statement

The data analyzed in this study is subject to the following licenses/restrictions: the data is part of the Danish national registry and cannot be disclosed. Requests to access these datasets should be directed to zeinab.schaefer@cs.au.dk.

## Author Contributions

ZS has contributed to the processing, analyzing the data, preparing the manuscript, discussion of results, revision of the manuscript, and the final approval. AM contributed to processing, design of the study, data visualization, preparing the manuscript, and discussion of results. SE, TR, and KK-M contributed to the idea and design of the study, discussions of the results, the revision of the manuscript, and final approval. KS contributed with data extraction and discussions regarding the results. All authors contributed to the article and approved the submitted version.

## Conflict of Interest

The authors declare that the research was conducted in the absence of any commercial or financial relationships that could be construed as a potential conflict of interest.

## Footnotes

^1^The project was approved by Statistic Denmark as project number 707670.
