# Supplementary material for: Toward Machine-Learning-Based Decision Support in Diabetes Care: A Risk Stratification Study on Diabetic Foot Ulcer and Amputation
Source: Front Med (Lausanne). 2021 Feb 18;7:601602. doi: 10.3389/fmed.2020.601602 (PMC7931152; doi:10.3389/fmed.2020.601602)
Supplement: Supplementary file 1 [file Data_Sheet_1.pdf]

1 APPENDIX

1.1 Codes

In this section all ICD-10 and ATC codes that have been used in this study are tabulated.

Medical Condition	ICD-10 codes
Diabetes Type I	DE10
Diabetes Type II	DE11
Diabetes*	DE (10-14)
Foot ulcer	DE(10-14)5B, DL89, DL97, DL984
Above knee amput (aka)	KNFQ (09,19,99)
Below knee amput (bka)	KNGQ(09,19,99)
Toe amput (ta)	KNHQ(1,2,11,14,17,20-25,27,99)
Foot amput (fa)	KNHQ (00,02,03,05,07)
Amputation	aka, bka, ta, fa

Supplementary Table 1. The ICD-10 codes used for determining diabetic patients in this study. We take all different diabetes types which is the list of codes in Diabetes*.

Medical Conditions	ICD-10 codes
Cardiovascular diseases	DI 20-25, DE105, DE115, DE125, DE135, DE145
Peripheral Artery diseases	DI73 (with all its subexpansions)
Hyperlipidemia	DE(780-786)
Hypertension	DI1 (with all its subexpansions)
Nervous system disorder	DG (with all its subexpansions)
Chronic Renal Complication	DN18(1-9) and DE(10-14)2
Neuropathy	DE104, DE114, DE124, DE134, DE144, DG730, DG990, DG590, DG632
Retinopathy	DE103, DE113, DE123, DE133, DE143
Mental diseases	DF (with all its subexpansions)
Dementia with Alzheimer	DF00 (with all its subexpansions)
Periodic Depression	DF33 (with all its subexpansions)
Urinary tract infection	DN109, DN300, DN309, DN390
Bone fracture	DS(0-9)2, DT0(2,8,10,12), DM483, DM484, DM485, DM843

Supplementary Table 2. The ICD-10 codes of different medical conditions that have been assessed in this study.

Medication Type	ATC codes
Diabetes	A10AB, A10BD, A10BX, A10BB
Cholestrol lowering drugs	C10AA

Supplementary Table 3. ATC codes used in this study.

1.2 Predictor model

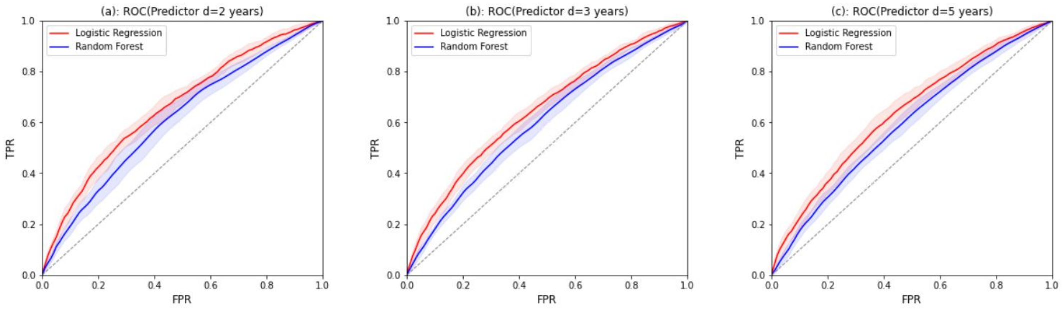

(a) Amputation with DFU

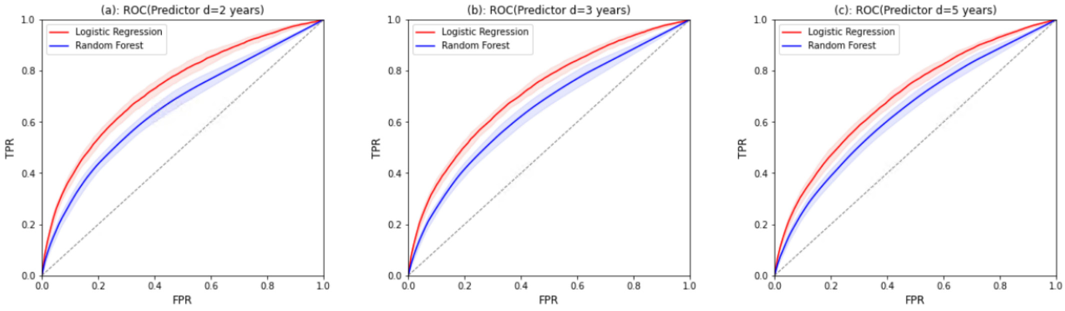

(b) DFU prediction

Supplementary Figure 1. ROC curves for prediction of (a) amputation for patients that have DFU in the next $d = 2, 3, 5$ years since the target year and (b) DFU occurrence.

1.3 Log hazard ratios

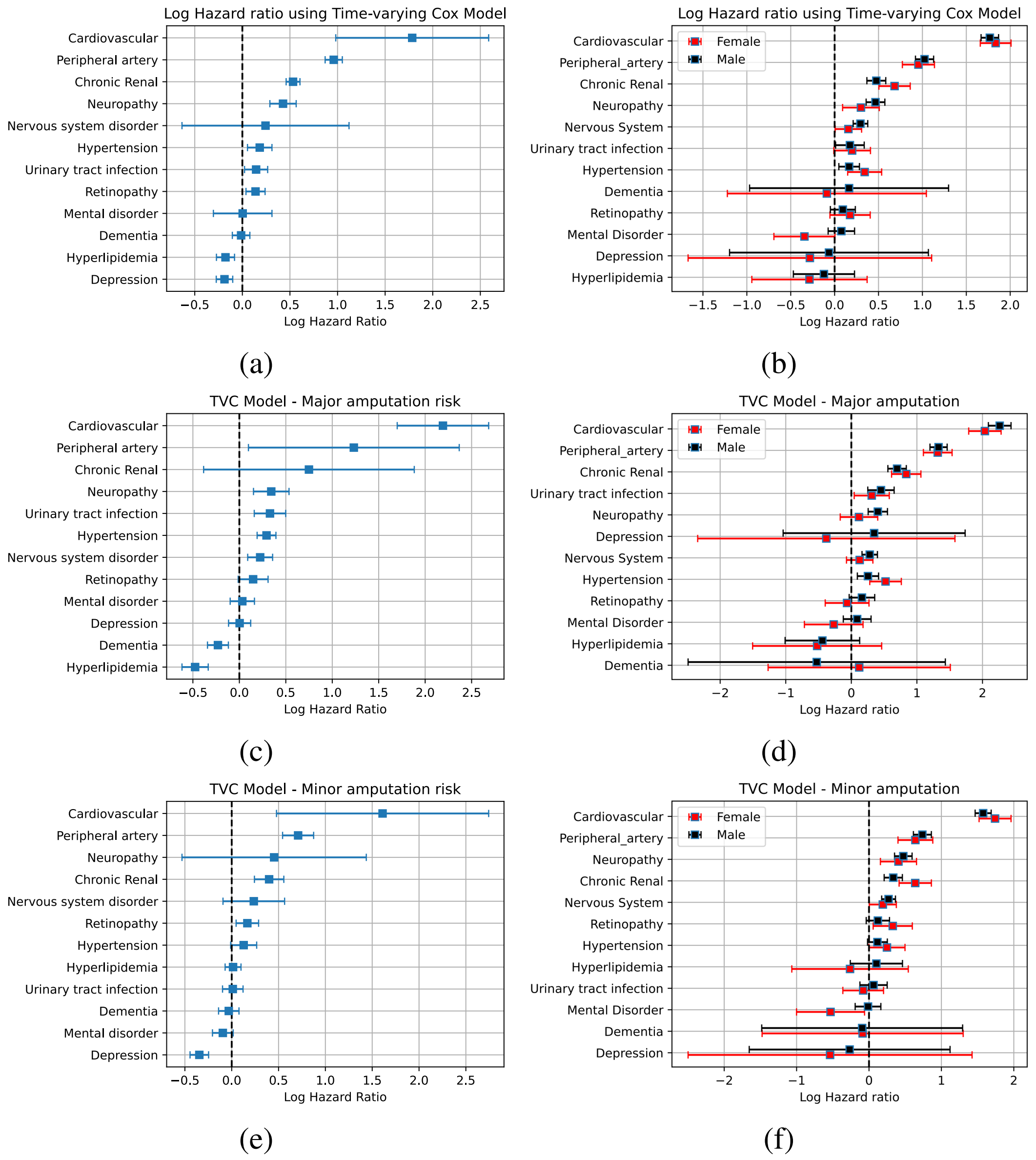

Supplementary Figure 2. Plots (a,b): risk of amputation for patients who have foot ulcer during the study timeline. Plots (c,d) and (e,f): Risk of major/minor amputation for patients with diabetes and foot ulcer respectively.

1.4 Aalen Johansen Model on Medical Conditions

Supplementary Figures 4 and 3 show the mortality risk and amputation for patients with diabetes who have one of the specific medical condition. Fig. show the mortality risk versus the risk of developing foot ulcer for patients with diabetes and specific medical conditions. Supplementary Figure 5 presents the risk of foot ulcer versus mortality.

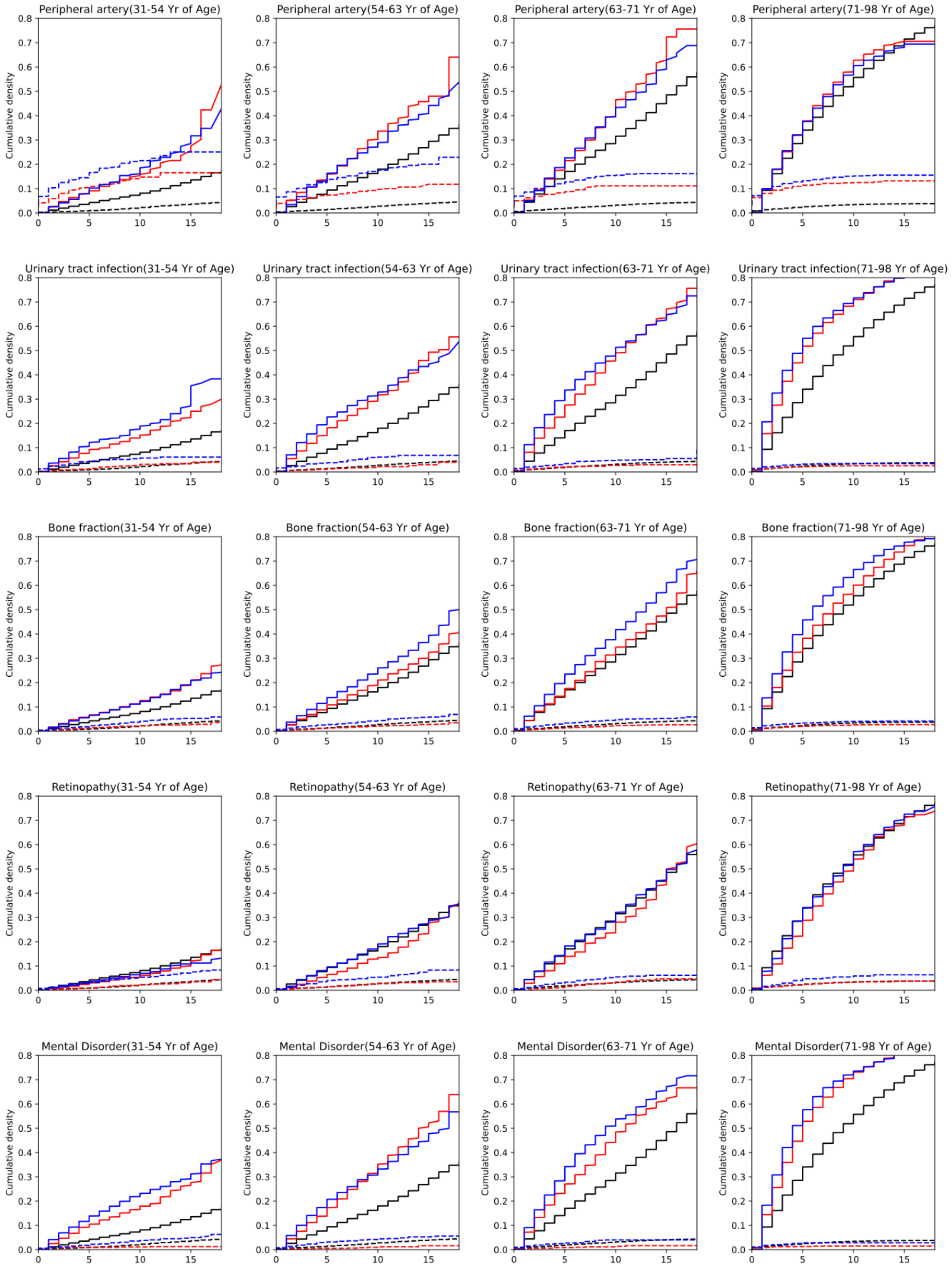

Supplementary Figure 3. Risk of developing amputation or death having diabetes and one of the medical conditions (Hypertension, Hyperlipidemia). Red/Blue/Black dashed: (Female/Male/Baseline) risk of developing amputation. Red/Blue/Black solid: (Female/Male/Baseline) risk of death.

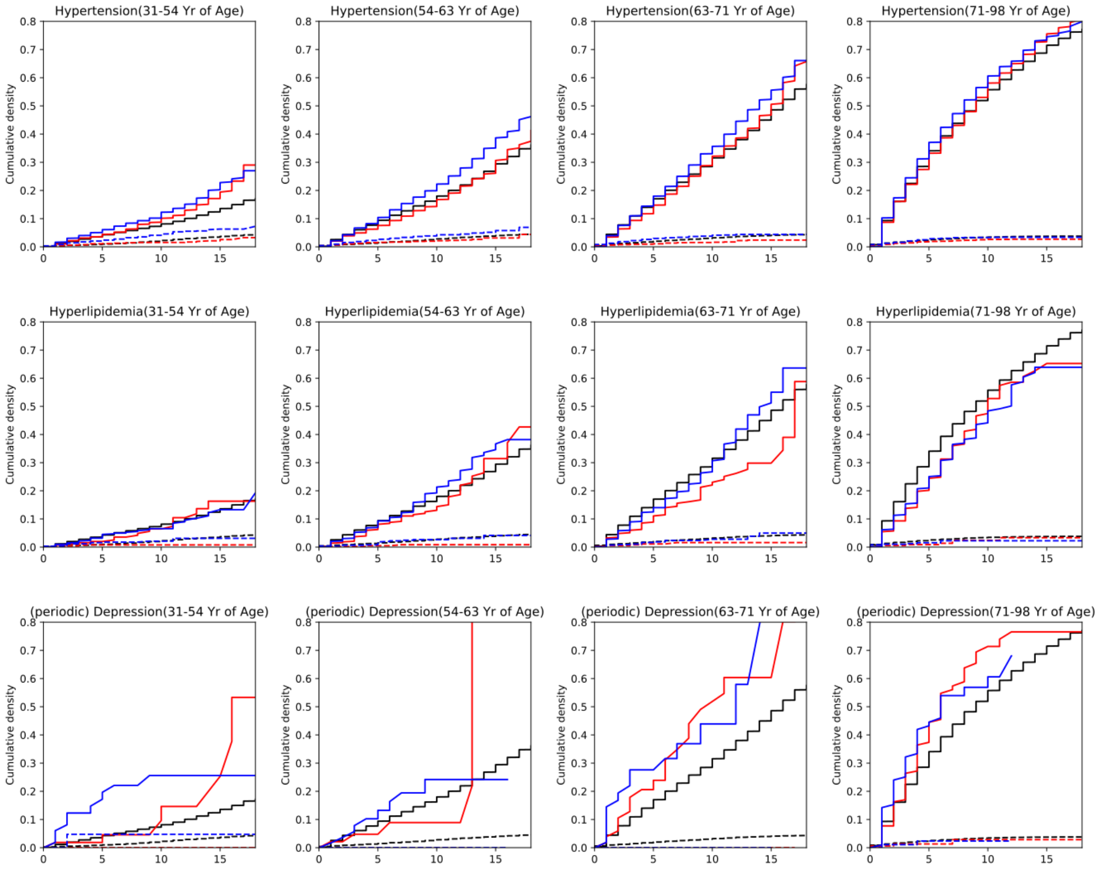

Supplementary Figure 4. Risk of developing amputation or death having diabetes and one of the medical conditions stated in the plots Red/Blue/Black dashed: (Female/Male/Baseline) risk of developing amputation. Red/Blue/Black solid: (Female/Male/Baseline) risk of mortality.

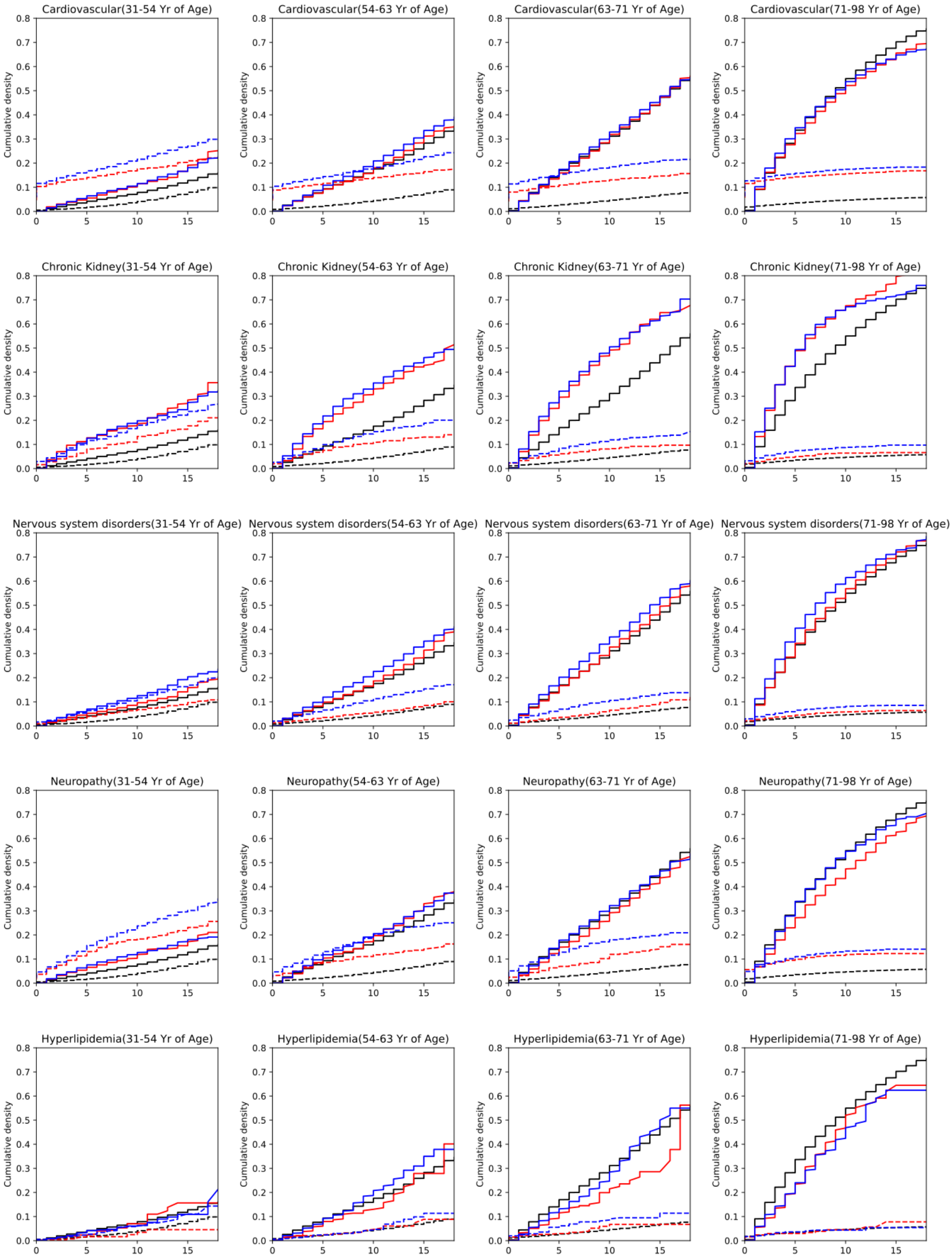

Supplementary Figure 5. Risk of developing foot ulcer or death having diabetes and one of the medical conditions (Hypertension, Hyperlipidemia). Red/Blue/Black dashed: (Female/Male/Baseline) risk of developing foot ulcer. Red/Blue/Black solid: (Female/Male/Baseline) risk of death.
